# Navigation in Unknown Dynamic Environments Based on Deep Reinforcement Learning

**DOI:** 10.3390/s19183837

**Published:** 2019-09-05

**Authors:** Junjie Zeng, Rusheng Ju, Long Qin, Yue Hu, Quanjun Yin, Cong Hu

**Affiliations:** College of Systems Engineering, National University of Defense Technology, Changsha 410073, China (J.Z.) (L.Q.) (Y.H.) (Q.Y.) (C.H.)

**Keywords:** autonomous navigation, unknown environments, deep reinforcement learning, continuous control

## Abstract

In this paper, we propose a novel Deep Reinforcement Learning (DRL) algorithm which can navigate non-holonomic robots with continuous control in an unknown dynamic environment with moving obstacles. We call the approach MK-A3C (Memory and Knowledge-based Asynchronous Advantage Actor-Critic) for short. As its first component, MK-A3C builds a GRU-based memory neural network to enhance the robot’s capability for temporal reasoning. Robots without it tend to suffer from a lack of rationality in face of incomplete and noisy estimations for complex environments. Additionally, robots with certain memory ability endowed by MK-A3C can avoid local minima traps by estimating the environmental model. Secondly, MK-A3C combines the domain knowledge-based reward function and the transfer learning-based training task architecture, which can solve the non-convergence policies problems caused by sparse reward. These improvements of MK-A3C can efficiently navigate robots in unknown dynamic environments, and satisfy kinetic constraints while handling moving objects. Simulation experiments show that compared with existing methods, MK-A3C can realize successful robotic navigation in unknown and challenging environments by outputting continuous acceleration commands.

## 1. Introduction

Autonomous navigation plays an important role in the fields of video games [1] and robotics [2], to generate reasonable trajectories for Non-Player Characters (NPCs) and meet the fundamental needs of mobility for real-life robots. This paper focuses on the navigation problem of non-holonomic robots with continuous motion control in unknown dynamic environments.

Existing approaches [3] can be divided into two categories: (1) those based on global environmental data; (2) those using only local environmental information. Algorithms from the first category usually conduct efficient path search in a heuristic or non-heuristic manner knowing precise environmental states. They include Simple Subgoal Graph (SSG) [4] and its variants [5], sampling-based path planning algorithms (such as Probabilistic RoadMaps (PRM) [6], Rapidly exploring Random Trees (RRT) [7]), etc. The sampling-based algorithms commonly used in robotics are complete and efficient. However, these methods need a model of the entire map often represented by grids or topology maps.

Methods in the second category use local environment data detected by sensors to plan motions for robots, which can avoid first-move lags [8] and have the potential to realize real-time planning. These algorithms are classified into non-learning-based and learning-based methods. Typical non-learning-based algorithms, such as Artificial Potential Fields (APF) [9] and Velocity Obstacle (VO) [10], calculate motion behaviors at every time step following preset rules. Their generated reactive strategies usually lead to bad performance in local minima areas. Besides, some key parameters of the algorithms are difficult to be manually adjusted, and the algorithms require some overly strict assumptions.

Local learning-based navigation algorithms include Deep Learning (DL) and Deep Reinforcement Learning (DRL). Methods based on DL [11] use deep neural networks to extract patterns of reasonable navigation behavior from a large number of labelled expert data. However, it is time-consuming and energy-consuming to collect labelled samples for navigation in unknown environments, which prevents DL-based methods from being widely applied to solve the proposed problem. In contrast, DRL cannot learn from labelled data but experience generated from interactions between the agent and the environment [12]. Generally, they can be divided into two sorts, namely value-based and policy-based DRL. Compared with value-based DRL methods, the latter methods are more competent to deal with continuous action spaces. Among them, Asynchronous Advantage Actor-Critic (A3C) [13] is well-received in the field of computer games [14,15] and robotics [16,17] owing to its high training efficiency. Nonetheless, applying A3C to solve the navigation problem in unknown dynamic environments is still facing the following challenges.

First, in an unknown environment the robot can only collect incomplete and noisy environmental data within a certain range through its own sensors, which make the robot controlled by A3C fall into traps of local minima at areas of concave canyon, long corridors and other challenging terrains. Other DRL algorithms are also difficult to avoid local minima. Second, for in most cases there is only one goal location, the rewards are sparsely distributed in the environment, which empirically greatly slows down the learning efficiency and even leads to non-convergence.

To solve the above problems, this paper proposes MK-A3C, a novel DRL algorithm that improves on the A3C algorithm by using memory mechanism, domain knowledge and transfer learning. MK-A3C constructs a Gated Recurrent Unit (GRU)-based network [18] architecture to store abstract historical information in the internal state of the GRU layer. In addition, MK-A3C introduces domain knowledge by reward shaping [19] and transfer learning to increase learning efficiencies and enhance generality. To deal with incomplete states, MK-A3C constructs the belief state by combining the abstracted historical trajectories with the current observation. Then, to overcome the challenge of sparse reward, MK-A3C builds a domain knowledge-based reward function and a transfer learning-based training task framework [20], which can increase the density of reward signals and meanwhile realize the migration of knowledge from simple tasks to complex tasks.

The rest of this paper is organized as follows: Section 2 discusses some related work about non-learning-based navigation methods, DL, and DRL. Section 3 presents our navigation approach in detail. In Section 4, the performance of the proposed method is evaluated through simulation experiments. Finally, Section 5 gives our conclusions.

## 2. Related Work

This section introduces related works in terms of navigation approaches including non-learning-based methods, DL and DRL. Generally, APF and velocity-based methods are typical non-learning-based navigation methods that are not suitable to deal with changes in unknown environments. Learning-based methods such as DL and DRL can enhance the adaptability of mobile robots in unknown environments by accumulating navigation experience. While DL requires large-scale labelled data that are difficult to collect, DRL merely needs data from interactions between agents and environments.

### 2.1. Non-Learning-Based Methods

It is difficult for non-learning-based methods to deal with navigation in unknown dynamic environments, since these methods cannot address unpredictable situations and is not adaptive to environmental changes. This subsection focuses on two typical methods, VO and APF. VO [10] can generate reachable trajectories by computing a velocity space of a robot. However, it is inefficient for real-time applications to compute maneuvers via the velocity space. Comparatively, APF [9] employs time-varying potential field functions as a low-cost navigation technique in dynamic environments, which uses only local information near the agent as input. Unfortunately, potential field functions, key to APF, are subject to several parameters, such as the relative strength between the repulsive and attractive potentials, which are generally determined by experience and easily incur local minima issues. In addition, non-learning-based methods generally make plans by current observations, thereby only finding reactive policies with poor adaptability to unknown and dynamic environments.

### 2.2. Deep Learning

DL is essentially a collection of models for non-linear function approximations through abstracting features hidden in inputs by deep neural networks and presently is widely used in robotic applications, such as robot manipulation, indoor navigation and so on [21,22,23]. Generally, navigation methods based on DL are included in two genres, i.e., data-driven control methods and Model-Predictive Control (MPC) methods. The first kind directly maps raw sensory inputs to control commands by large-scale supervised learning. For example, Xu et al. [21] developed an end-to-end trainable architecture to learn a generic vehicle motion model based on a large set of crowd-sourced video data. Tai et al. [22] adopted a convolutional neural network (CNN) to learn indoor navigation policies by self-constructed indoor depth data sets. The second kind of approach uses deep neural networks to learn dynamics of controlled agents, and then calculates optimal control input based on the predicted future outputs. For instance, Agrawal et al. [23] took raw sensory images as input to predict the dynamic model of robotic arms for manipulation by deep neural networks. However, it is difficult to collect a large set of labelled data for training deep neural networks in the scenario of navigation in unknown environments.

### 2.3. Deep Reinforcement Learning

In contrast to DL-based methods, DRL can learn navigation policies by samples collected from interactions between the agent and the environment. Therefore, DRL can avoid collecting large-scale labelled data. In addition, unlike DL which extracts existing navigation patterns from data sets, DRL can explore unseen policies by actively interacting with the environment, which brings about further breakthroughs in autonomous navigation. Currently, much research is devoted to solving problems of navigation in static unknown environments. For example, Tai et al. [24] proposed asynchronous deep deterministic policy gradients to learn navigation policies under simple static unknown environments. Wang et al. [25] proposed a fast-recurrent deterministic policy gradient algorithm (fast-RDPG) to accomplish unmanned aerial vehicles’ navigation tasks in more complex environments. Zhelo et al. [26] proposed curiosity-driven exploration strategies to argument robots’ abilities to explore complex unknown environments. However, these works mainly focus on navigation in static environments, which are unpractical for most realistic applications since various kinds of moving objects are required to be taken into consideration.

Compared with the above methods, MK-A3C can solve the navigation problem in unknown dynamic environments with moving obstacles by introducing memory mechanism and domain knowledge into its DRL framework.

## 3. The Methodology

In this section, we first introduce the navigation problem and related backgrounds, and then build a model for the crawler robot. Then, we illustrate the architecture of the proposed algorithm. Finally, the motion planner based on MK-A3C is described in detail.

### 3.1. Problem Description

The targeted navigation problem in this paper can be described as follows: In an initially unknown environment where terrains like dead corners and long corridors are widely distributed, a mobile robot only equipped with sparse local distance sensors is required to move safely to a given destination controlled by continuous control commands. Since the environment is initially unknown, the robot can only build its own environmental model through exploration. Meanwhile, the environment with moving obstacles is dynamic and uncertain, which greatly increases the complexity. As proved in reference [27], navigation in dynamic environments is a non-deterministic polynomial-hard problem.

**Figure 1:**
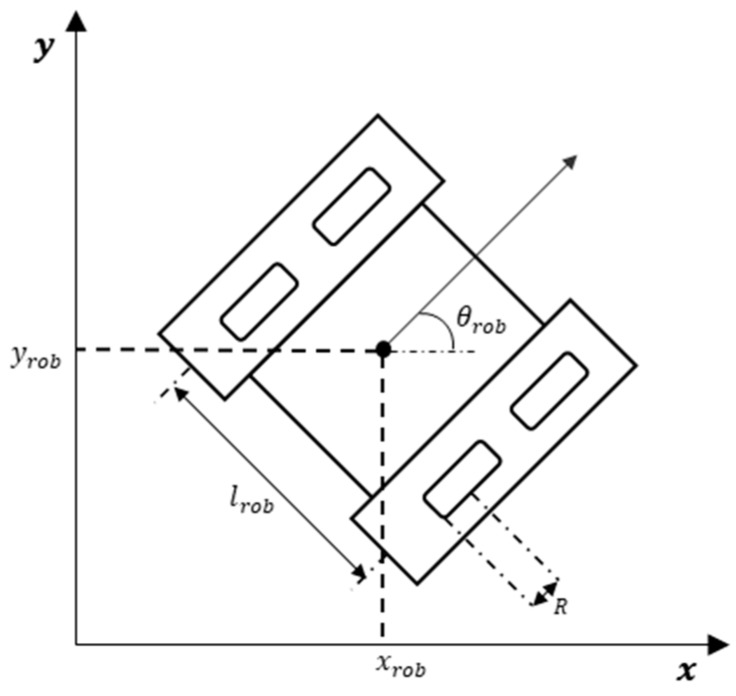
Schematic diagram of the crawler robot.

In this paper, we adopt a Cartesian coordinate system. The model of the mobile robot is illustrated in Figure 1 and its dynamics are given in Equations (1) and (2). We use a tuple $\left(x_{rob},y_{rob},\theta_{rob}\right)$ to denote the position information of the mobile robot, where $x_{rob}$ and $y_{rob}$ are the robot’s global horizontal and vertical coordinate respectively, and $\theta_{rob}$ is the angle between the orientation of the robot and the positive abscissa. To decrease the hardware requirements and take moving obstacles into consideration, the range sensor equipped with the robot has a 360-degree detection angle range, and the detected angular interval is 15 degrees, which means that 24-dimensional local obstacle distance information can be obtained. Its maximum detection distance of the sensor is 7 m. The mobile robot is a crawler robot that can be controlled by the left and right tracks. To achieve meticulous manipulations and meet kinetic constraints, continuous angular acceleration is used as control commands. The kinetic equation is described as follows:

(1) $$\left(\begin{array}{c} \begin{array}{c} {\dot{x}}_{rob}\\ {\dot{y}}_{rob} \end{array}\\ {\dot{\theta }}_{rob} \end{array}\right)=R\left[\begin{array}{cc} \frac{cos\theta_{rob}}{2} & \frac{cos\theta_{rob}}{2}\\ \frac{sin\theta_{rob}}{2} & \frac{sin\theta_{rob}}{2}\\ \frac{1}{l_{rob}} & \frac{-1}{l_{rob}} \end{array}\right]\left(\begin{array}{c} \omega_{l}\\ \omega_{r} \end{array}\right),$$

(2) $$\left(\begin{array}{c} {\dot{\omega }}_{l}\\ {\dot{\omega }}_{r} \end{array}\right)=\left(\begin{array}{c} \frac{\partial \omega_{l}}{\partial t}\\ \frac{\partial \omega_{r}}{\partial t} \end{array}\right),$$

where R is the radius of the driving wheel; $\omega_{l}$ and $\omega_{r}$ denote the angular velocities of the left and right driving wheels respectively (rad·s^−1^); ${\dot{\omega }}_{l}$ and ${\dot{\omega }}_{r}$ denote the angular accelerations of them (rad s^−2^); $l_{rob}$ denotes the distance between two tracks.

### 3.2. The Architecture of the Proposed Algorithm

**Figure 2:**
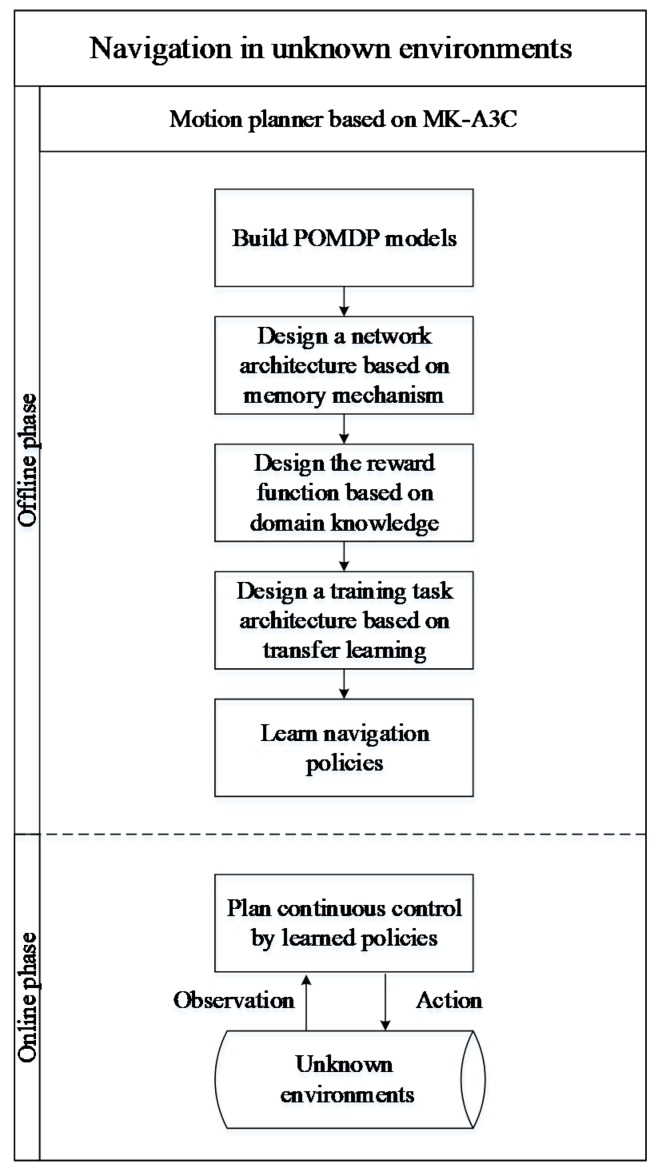
Flowchart of the MK-A3C method.

In this paper, the proposed MK-A3C algorithm makes improvements on the original A3C algorithm by adding the memory mechanism and domain knowledge. In addition, MK-A3C can avoid the local minima trap caused by partial observability and the sparse reward challenge in unknown environments. Figure 2 shows the architecture of the proposed algorithm.

As shown in Figure 2, in the offline phase of the motion planner, the main task is to learn navigation policies in unknown dynamic environments. First, key information about the robot and the environment is extracted to construct a Partially Observable Markov Decision Process (POMDP) model, since in unknown environments the robot cannot directly obtain the complete environmental state to construct a general Markov Decision Process (MDP) model. Then, a neural network architecture with a memory mechanism is built to solve the POMDP model, which integrates historical data with the current observations to construct the robot’s own belief state as its cognition about environments. Next, to overcome the challenge of sparse reward, a reward function based on domain knowledge is constructed by reward shaping, which can increase the density of reward signals and accelerate the convergence of the training process. To further improve learning efficiencies, the architecture of training tasks is constructed by transfer learning. Finally, MK-A3C is used to learn the navigation policy. In the online phase, navigation policy learned in the offline phase takes the environmental information detected by the sensor and internal states of the robot as its input, and output continuous control commands to navigate the robot to a given goal location.

MK-A3C can learn near-optimal policies to navigate robots in complex unknown environments and satisfy kinetics and task-specific constraints. The algorithm embraces two main features. The first is that it builds a novel DRL framework to address partial observable problems. Specifically, the memory-based neural network integrates historical information and current observations to solve the POMDP that formally describes the targeted navigation problem, which equips the robot with temporal reasoning ability. Additionally, the neural network based on GRU contains the robot’s cognition of the environment and can be used to avoid being trapped in local minima.

Second, an innovative reward function and the novel architecture of training tasks are constructed to training the memory network. Specifically, we introduce domain knowledge into the reward function by reward shaping to solve the problem of sparse rewards. The shaped reward function can increase the destiny of reward signals to accelerate the training process. Besides, inspired by reference [17], an architecture of training tasks is built by transfer learning, which can improve training efficiencies by effectively using accumulated knowledge from the previous learning task. In conclusion, improvements to reward function and training architecture are helpful to collect effective training samples in unknown environments, thereby relieving the method from the problem of sparse reward. In addition, due to the generalization of MK-A3C, the motion planner can quickly adapt to new environments without retraining.

### 3.3. Motion Planner Based on MK-A3C

This section introduces the motion planner based on MK-A3C in the following four parts: (1) backgrounds and concepts of POMDP; (2) description of the proposed POMDP; (3) basic concepts of policy-based DRL methods and A3C algorithm; and (4) a description of the MK-A3C algorithm.

#### 3.3.1. Backgrounds and Concepts of POMDP

Policy-based DRL algorithms, such as the Deep Deterministic Policy Gradient (DDPG) [28], A3C, and Distribute Proximal Policy Optimization (DPPO) [29,30], are powerful tools for solving MDP models. However, navigation in unknown environments cannot be formalized as an MDP model since the robot cannot obtain the complete state $s_{t}$. Specifically, the sensor mounted on the robot can only obtain distance information of nearby obstacles, and even cannot directly know velocities of moving obstacle and the global terrain. POMDPs, as an extension from the MDP models can describe uncertain interactions between robots and environments where only a limited range of area can be obtained. Therefore, we model the process of robots’ safe navigation in unknown dynamic environments as POMDPs, which can better capture the hidden state in the environment through the analysis of environmental observations.

Due to the terminal conditions in the proposed navigation problem, we use a finite POMDP that can be formally described as a 6-tuple (S,A,P,R,O,Ω). Among the elements, S is a finite set of states, A is a finite set of actions, O is a finite set of observations and P denotes a state transition function being part of the environment model. $P\left(s,a,s^{\prime }\right)=P[s_{t+1}=s^{\prime }|s_{t}=s,a_{t}=a]$, denotes the probability of transferring to the next state $s^{\prime }$ when the action a is executed under the current state s. Besides, $R\left(s,a,s^{\prime }\right)=f\left(s_{t}=s,a_{t}=a,s_{t+1}=s^{\prime }\right)$ is a reward function to quantify the immediate feedback from the environment when the agent takes action a in the current state s, indicating an evaluation of the agent’s behavior at the current step [31]. $\Omega \left(o^{\prime },s,a\right)=P[o_{t+1}=o^{\prime }|s_{t}=s,a_{t}=a]$, an observation function, denotes the probability of receiving to the next observation $o^{\prime }$ for the agent when the action a is executed in the current state s.

Although in unknown environments the robot cannot directly obtain the complete environmental state, in the POMDP model, the robot’s inner representation of the environment can be constructed through historical data, i.e., the belief state Bs. Due to the powerful feature extraction capability of the neural network, we choose the recurrent neural network unit GRU to construct the belief state. GRU takes the last belief state $Bs_{t-1}^{a}$, the current observation $o_{t}$ and last action $a_{t-1}$ as its input to estimate hidden environment states.

#### 3.3.2. Description of Navigation POMDP

It is difficult to construct models for unknown dynamic environments, which means that the state transition function P and the observation function Ω are unknown. The constructed POMDP model is presented in detail, including the observation space O, the action space A and the reward function R.

(3) $$O=\left(S_{1},S_{2},\ldots ,S_{24},d_{tar},a_{tar},\theta ,\omega_{l},\omega_{r}\right)$$

The observation space O consists of sensor data, the relative position between the target and the robot, and the internal state of the robot. The sensor data includes 24 dimensions distance information of nearby obstacles which cover 360-degree detection range at intervals of 15 degrees. To be specific, $S_{i}$ (i∈(1,2,3…,24)) refers to the distance to the nearest obstacle located in the range from (i−1)∗15 to i∗15 degrees. If no obstacle is detected, the value of $S_{i}$ is the maximum detection distance. The value range of $S_{i}$ is [0,7], whose unit is meter. Since the environment is dynamic, we abandon the mobile robot’s absolute position $\left(x_{rob},y_{rob},\theta_{rob}\right)$, and use the relative position to describe the relationship between the target and the robot. The relative position $\left(d_{tar},a_{tar}\right)$ includes the distance and orientation information between the target and the robot. $d_{tar}$ refers to the Euclidean distance between the robot and the destination and $a_{tar}$ represents to the angle between the robot’s orientation and the line connecting the robot and the target. If the target is on the left hand side of the robot, the value of $a_{tar}$ is set positive, otherwise negative. Its value ranges from [−π, π]. The internal information of the robot includes its orientation, angular velocities of its left and right driving wheels. θ refers to the orientation of the robot, and its value ranges from [0, 2π]. $\omega_{l}$ and $\omega_{r}$ refer to the angular velocity of the left and right driving wheels, ranging from [−0.5, 0.5] in rad$\cdot s^{-1}$.

(4) $$A=\left({\dot{\omega }}_{l},{\dot{\omega }}_{r}\right)$$

The action space A includes the angular accelerations of left and right driving wheels in tracks, ${\dot{\omega }}_{l}$ and ${\dot{\omega }}_{r}$. Range of their value is [−0.5, 0.5], and their unit is rad$\cdot s^{-2}$. In the problem of robot navigation, the output action is generally represented in the following two ways: (1) discrete actions, such as forward, left turn and right turn; (2) continuous velocities, such as linear velocity and steering velocity. To achieve more realistic and precise control, we take the continuous angular acceleration of the driving wheels as output.

The reward function R is an instant evaluation of the executed action’s effects in the current state. For a fully observable MDP model, the reward function can be generally set as obtaining 1 if realizing the goal and obtaining 0 if not. However, in the proposed partially observable problem, adopting the simple reward function definitely leads to sparse reward. Specifically, since the initial policy is randomly generated, the probability that the robot reaches the destination in an unknown dynamic environment with long corridors and dead corners is close to zero, which means that it is hard to collect effective samples. Reward shaping [19], as a way to solve sparse rewards, can provide early rewards for agents to accelerate the convergence of training policies while ensuring policy invariance.

We use reward shaping to embed the domain knowledge into the reward function. The shaped reward function is divided into two parts, i.e., non-terminal rewards and terminal rewards, respectively. Terminal rewards include rewards of reaching target and collision penalties. The first part of terminal rewards is described as follows:

(5) $$r_{arr}=10;\;if\;d_{t}<d_{tol}$$

where $d_{tol}$ refers to the tolerance distance to the target.

The equation of collision penalties is as follows:

(6) $$r_{col}=-5;\;if\;S_{min}<d_{saf}$$

where $S_{min}$ refers to the minimum obstacle distance; $d_{saf}$ refers to the safety distance between the robot and the obstacle. The idea behind the collision penalty, essentially a negative reward, is that when the distance between the robot and the obstacle is less than the safe distance, the robot will be at the risk of colliding with another object and failing in the task.

Non-terminal rewards include rewards for safe orientation and closer distance to the target and penalties for collision danger and time consumption. Their equations are as follows:

(7) $$\left\{\begin{array}{c} r_{s_{ori}}=0.002\\ r_{step}=0.001\\ r_{dang}=\eta *2^{\zeta d_{min}}\\ r_{d\_gol}=\lambda \left(d_{tar}{}_{t-1}-d_{tar}{}_{t}\right) \end{array}\right\}$$

Rewards for safe orientation denote that a smaller reward is gained when the minimum obstacle distance in the forward direction of the robot is greater than a given value. The design of this reward not only further ensures the safety of the navigation process, but also helps the robot to escape from the local minima trap. Rewards for closer distance to the target denote that when the robot gets closer to the target, it obtains a positive reward, otherwise obtains a negative reward. λ is a parameter used to control the strength of this reward.

Additionally, when the robot is closer to the obstacle, it will get more negative rewards, known as danger penalties. The reward signal can provide the robot with strong safety awareness. Time penalty denotes that during the training process, the robot receives a smaller negative reward at every step. This reward can encourage the robot to reach the destination in less time. In addition, hyper-parameters of reward function slightly change as the difficulty of the training task increases.

#### 3.3.3. A3C Method

In DRL, V(s) refers to the long-term expected cumulative reward starting from the state s, which is usually used to evaluate the state s. Q(s,a) denotes the long-term expected cumulative reward starting from executing action a in state s. Π(s,a) refers to the probability of executing action a under state s [31].

In this paper, we improve A3C algorithm to solve the proposed navigation problem for two reasons: (1) compared with value-based DRL methods such as Deep Q-Network (DQN) [32] and its various improved versions [33,34], A3C can deal with continuous action space and generate excellent policies; (2) compared with other policy-based DRL methods such as DDPG and Trust Region Policy Optimization (TRPO) [35], A3C consumes less GPU computation resources and training time. Besides, it is convenient to debug and optimize A3C method thanks to its clear and elegant structure.

Before proposing A3C, experience replay [32] was used to solve the non-convergence problem of combining deep neural network with traditional off-policy Reinforcement Learning (RL) methods. The experience replay method stores samples collected from interactions between the agent and the environment in the experience memory unit, and then randomly sample training data. However, this method is not suitable for on-policy RL methods, because samples used to train policies must be generated by this policy in this kind of methods. To combine on-policy RL methods with deep neural networks, Mnih et al. [13] proposed a simple lightweight framework that uses asynchronous gradient descent to optimize neural network controllers. The framework can be applied to the traditional on-policy Reinforcement Learning (RL) algorithm, actor-critic, known as A3C for short. In contrast to the experience replay method, the asynchronous framework reduces the correlation between training samples by asynchronously executing multiple agents simultaneously in multiple instances of the environment.

**Figure 3:**
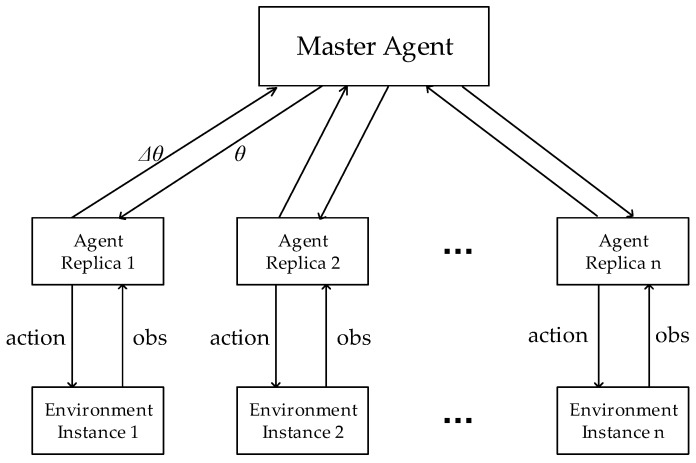
The diagram of Asynchronous Advantage Actor-Critic (A3C).

As shown in Figure 3, the core idea of A3C algorithm is to asynchronously execute multiple replicas of the agent in multiple environmental instances. During the training process, each of them interacts with its own environmental instance, collects different training samples, calculates the network’s gradient, uploads the gradient to update the global network, and finally downloads the latest network to the local. Please note that the master agent’s global network cannot be updated by multiple agent replicas’ uploaded gradients at the same time.

Consistent with the network design of the AC algorithm, A3C uses the actor network and the critic network to parameterize the policy function $\Pi (a_{t}|s_{t};\theta_{p})$ and the value function $V\left(s_{t};\theta_{v}\right)$, respectively. Each agent replica calculates gradients of actor and critic networks in every fixed step or at the end of the episode, and then uploads gradients asynchronously to update the global network. The gradient calculation formula of the critic network is as follows:

(8) $$d\theta_{v}=\partial {\left(A\left(s_{t};\theta_{v}\right)\right)}^{2}/\partial \theta_{v}$$

where $A\left(s_{t};\theta_{v}\right)$ denotes an estimate of the advantage function given by:

(9) $$A\left(s_{t};\theta_{v}\right)=\sum_{i=0}^{k-1}\gamma^{i}r_{t+i}+\gamma^{k}V\left(s_{t+k};\theta_{v}\right)-V\left(s_{t};\theta_{v}\right)$$

where γ is the discount factor and k is the difference between the length of the sample sequence and the index of the current sample (coded from zero).

The gradient calculation equation of the actor network is as follows:

(10) $$d\theta_{p}=\nabla_{\theta_{p}}log\Pi \left(a_{t}|s_{t};\theta_{p}\right)A\left(s_{t};\theta_{v}\right)+\beta \nabla_{\theta_{p}}H\left(\Pi \left(s_{t};\theta_{p}\right)\right)$$

where H is the entropy of the policy Π. The hyper-parameter β controls the strength of the entropy regularization term. Adding entropy to the process of updating the policy function’s gradient can improve the agent’s exploration capability and prevent policies from converging to the local optimal solution [13].

#### 3.3.4. MK-A3C Method

Directly applying A3C to solve navigation problems in unknown dynamic environments faces challenges of sparse rewards and incomplete states. In Section 3.3.1, to solve the problem of non-convergence policies caused by sparse reward, we use reward shaping to construct reward functions with domain knowledge. In this section, to further overcome above challenges, improvements are made on A3C algorithm to: (1) construct a memory network architecture based on GRU; (2) and build a training task architecture based on transfer learning.

**Figure 4:**
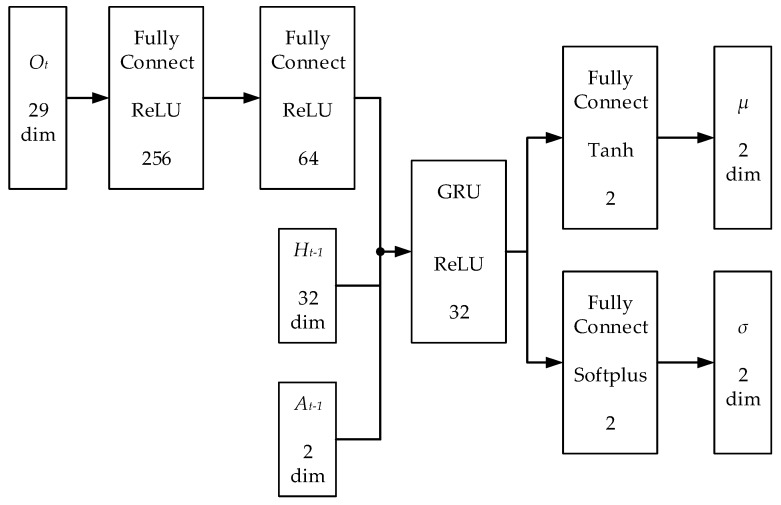
The structure of actor network in MK-A3C. $O_{t}$ denotes the current observation. $H_{t-1}$ is the last hidden states of GRU layer. $A_{t-1}$ denotes last action. μ denotes expectation of the output action probability distribution, and σ denotes variance. The structure of critic network is similar to that of actor network, but replaces two streams of the output layer of the actor network with one stream and no activation function.

Firstly, the GRU-based memory network architecture can avoid being trapped in local minima caused by partial observability of environments. A3C is used to solve MDPs, but MDPs can only describe fully observable problems such as Go, Chess, etc. Therefore, a POMDP model is built to formally describe the proposed problem. To solve the POMDP, this paper constructs a GRU-based memory network architecture. The recurrent neural network unit GRU is used as a function approximator of the belief state $Bs\left(o_{t}\right)$, which takes abstract historical data, the current observation and last action as input. As shown in Figure 4, the observation $o_{t}$ first is passed through two layers of Fully Connected (FC) layers, and the numbers of their hidden units are 256 and 64, respectively. Then, the GRU layer takes them as input that are extracted from 64 dimensions of observation features, the 32 dimensions of the last hidden state of the GRU and the two dimensions of the last action. The number of hidden units of the GRU layer is 32. The output layer is divided into two streams. One stream uses a FC layer to output the expectation of the Gaussian distribution of the action space, and the other stream outputs the variance. Instead of directly outputting actions, constructing the action distribution can improve the randomness of the action selection in the training process and increase explorations in unknown environments.

In the proposed network, the first two FC layers are used to extract features from the current observation. The GRU layer with recurrent mechanism is used to approximate the belief state by integrating features of the current observation with abstract historical trajectories. Finally, a FC layer is used to output the action distribution. The key to realizing memory and inference is to use the last hidden state of GRU layer as part of the current input of GRU layer, which means that GRU layer can use the historical trajectory as input to infer the current complete state (i.e., $input_{gru}^{t}=output_{gru}^{t-1}+a_{t-1}+output_{fc\_2}^{t}$, or $input_{gru}^{t}=f(o_{t},a_{t-1},o_{t-1},\ldots ,o_{0}$)).

Secondly, although reward shaping is used to introduce domain knowledge into the reward function, it is still difficult for the randomly initialized policy to collect valid samples in complex navigation tasks. To solve this problem, a training task architecture is constructed by transfer learning which can realize the migration of knowledge between different learning tasks. Specifically, this paper uses the curriculum learning method to build the architecture of training tasks whose complexity gradually increases. Curriculum learning [36] is a method that gradually increases the complexity of training samples to accelerate the training process. Although the method was originally proposed to solve problems of supervised learning, this method can also be applied to solve the challenge of sparse reward encountered by RL approaches. Since the network structure contains the recurrent network unit GRU, it is not appropriate to directly use the original curriculum learning. Inspired by reference [37], we propose an improved curriculum learning method to train GRU-based memory network architecture, which divides the whole learning task into two parts. The complexity of the first part increases with training episodes, while the complexity of the second part is randomly determined, but the minimum value of its complexity is greater than the complexity of the first part. In this paper, two indicators, i.e., the number of moving obstacles and the initial Euclidean distance between the robot and the destination, are used to quantify the complexity of the learning task. Key parameters of the first part of learning tasks are presented in Table 1.

In conclusion, MK-A3C solves problems of incomplete states and sparse reward by memory mechanism and domain knowledge, which can navigate the non-holonomic robots in unknown dynamic environments.

## 4. Experiments and Results

To verify the effectiveness of the MK-A3C algorithm, we implement mentioned improved components including the GRU-based memory network architecture, the shaped reward function with domain knowledge and the transfer learning-based training task architecture. To evaluate the performance of MK-A3C, we test it in unknown dynamic environments with moving obstacles. The motion pattern of the moving obstacles is the uniform linear motion, imitating common human movement behaviors.

In the simulation environment, numerical methods are used to calculate trajectories of the robot by kinetic equations. Due to limitations of the hardware platform, the step of simulation time is set as 0.1 s by considering both efficiencies of calculation and accuracies of results.

The hyper-parameters of MK-A3C are described as follows: the learning rates of actor network and critic network are 0.00003 and 0.0003, respectively. The unrolling step is 10. The discount factor γ is 0.99. The goal tolerance distance is 0.7. In the training phase, the action selection interval is 2 s, while in the test phase, it is 1 s.

Experiments are carried out on a machine with 3.4-GHz Intel Core i7-6700 CPU and 16 GB of RAM.

### 4.1. Training Environment Settings

**Figure 5:**
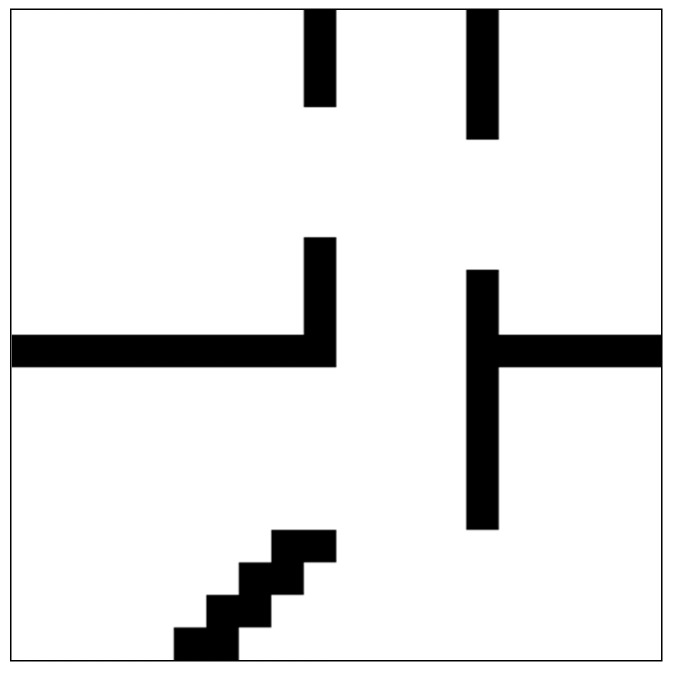
Training environment. Black areas are occupied by obstacles, and white areas are traversable for robots.

As shown in Figure 5, the training environment is a complex environment filled with long corridors and dead corners, the size of which is 20 by 20. In each episode, the starting point is randomly sampled from the lower-left corner of the map, and the goal point is randomly initialized within a certain range from the starting point. The initial motion states of moving obstacles are also randomly initialized in each episode. The range of velocities of moving obstacles is [0.06, 0.08]. To increase the difficultly of navigation problem, the initial directions of moving obstacles are roughly toward the robot. If moving obstacles reach the border of the map or collide with static obstacles, they will be initialized again. When the robot reaches the goal or collides with obstacles, the current episode ends and the new episode begins.

### 4.2. Evaluation of MK-A3C

To verify the effectiveness of the GRU-based memory network, we compare MK-A3C with A3C-R. A3C-R replaces the GRU layer in MK-A3C with a FC layer and removes the input of last action and abstract history data. The reward function and the training task architecture of A3C-R are consistent with MK-A3C. The value function V(s) refers to the total long-term expected discount rewards starting from state s, which can be used to reflect the performance of the learned policy. The success rate denotes the probability that the robot safely reaches the goal point in the nearest 1000 episodes.

**Figure 6:**
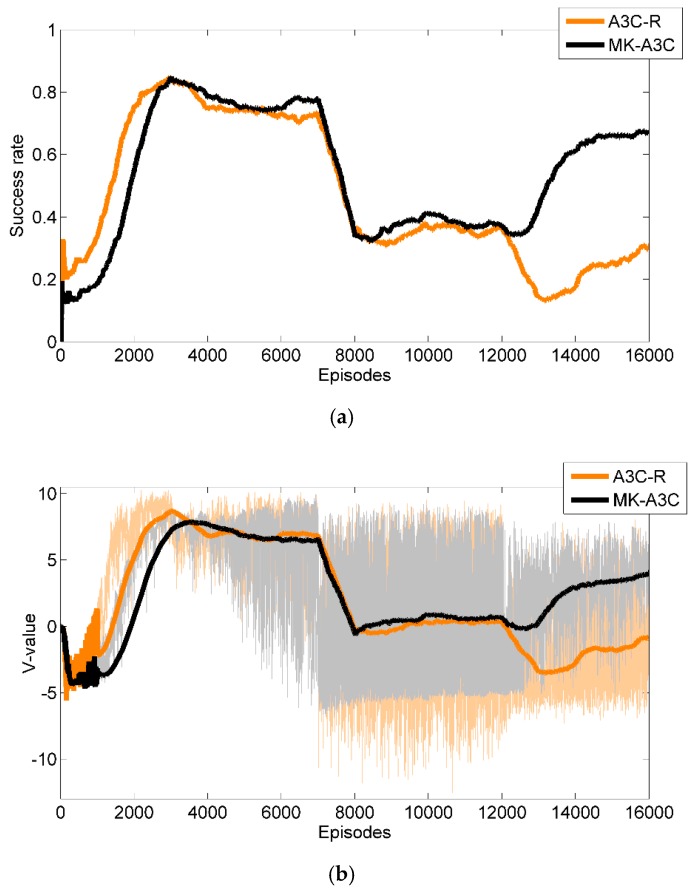
The performance of our method MK-A3C and A3C-R in the training phase. (**a**) The orange and black lines denote success rates of A3C-R and MK-A3C, respectively; (**b**) The light orange and gray lines represent data of *V*-value, and the overstriking orange and black lines denote the mean *V*-value of the last 1000 episodes.

As shown in Figure 6a, when the number of episodes is less than 3000, the performance of A3C-R is slightly better than that of MK-A3C. It shows that the A3C-R can handle simple navigation tasks well, and the learning efficiency of GRU-based memory network is lower than that of FC-based networks. When they collect experience from more episodes, until 12,000, the performance of MK-A3C is slightly better than that of A3C-R, and trends showed by the two curves are similar. When the number of episode is greater than 12,000, MK-A3C can have more successful navigation instances than its counterpart. This proves that the reactive navigation policies generated by A3C-R cannot deal with complex navigation tasks by only considering the current observation, while MK-A3C estimates hidden environmental states by combining historical trajectories and the current observation and is capable of finding more reasonable navigation policies. In addition, the training task architecture based on transfer learning is also more suitable to train the GRU-based network. In Figure 6b, their trends of *V*-value data in the training process are the same as that of success rate curves, which further proves the previous conclusions.

To evaluate the validity of the domain knowledge-based reward function constructed by reward shaping, we remove the non-terminal rewards from MK-A3C’s reward function. However, MK-A3C without non-terminal rewards cannot converge. MK-A3C without the proposed training architecture cannot converge to useful policies, which demonstrates that the transfer learning-based training task architecture is necessary for MK-A3C to solve navigation in unknown environments. In conclusion, experiments on the reward function and the training task architecture show that improvements to these two components can solve the problem of sparse reward by increasing the density of reward signals and improving learning efficiencies.

### 4.3. Performance on Unknown Dynamic Environments

In this section, to fully evaluate performance of the proposed method, we design more dynamic testing environments than that used for training, and their topologies are also very different. Specifically, the distributions of traversable areas are different among testing environments. The shapes and positions of static obstacles are randomly chosen. Besides, starting points are randomly sampled from the lower-left corner of the environment, and goal points are randomly distributed in the environment.

To increase dynamics of testing environments, we add disturbance in the positions, orientations and velocities of moving obstacles. The moving obstacles are randomly distributed in environments and their orientations are randomly selected from 0 to 360 degrees. The velocities are randomly chosen from 0.06 to 0.08. The uniform linear motion is the motion pattern of moving obstacles. There are five moving obstacles in the testing environments, which is more than that in the training phase.

**Figure 7:**
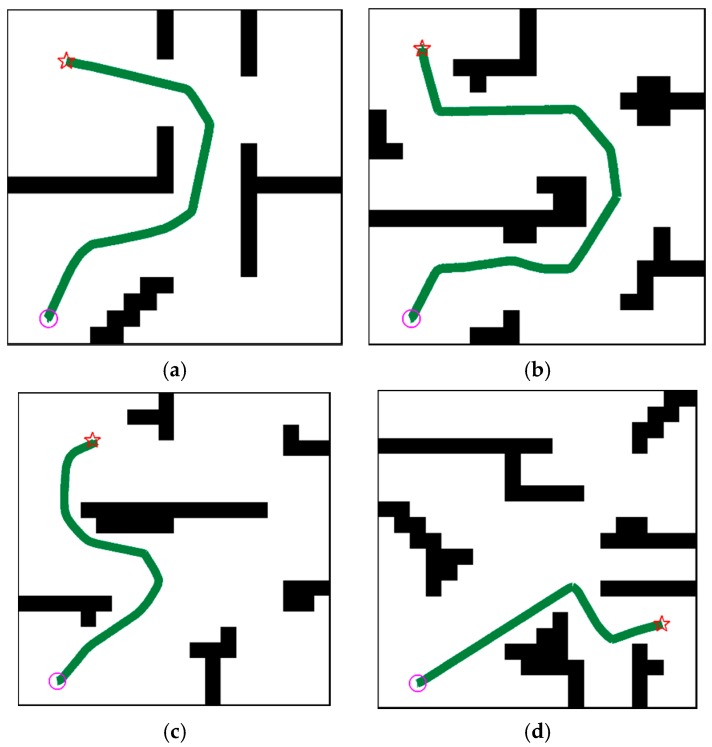
Examples of the robot completing one navigation tasks in the training and testing environments. The light purple circle denotes the starting point and the red pentagram denotes the goal point. The dark green lines are the trajectories generated by MK-A3C. For clear presentation, we do not show trajectories of moving obstacles. (**a**) Training Environment; (**b**) Testing Environment 1; (**c**) Testing Environment 2; (**d**) Testing Environment 3.

The performance of the MK-A3C is evaluated on tasks of navigation in unknown dynamic environments. As shown in Figure 7, the MK-A3Cbased motion controller is capable of implementing efficient and safe navigation in unknown challenging environments with moving obstacles. Besides, navigation policies generated by MK-A3C have powerful generalization capabilities, and can adapt to new unknown environments without retraining. Since A3C-R cannot complete all difficult examples in Figure 7, we do not present paths generated by A3C-R.

**Figure 8:**
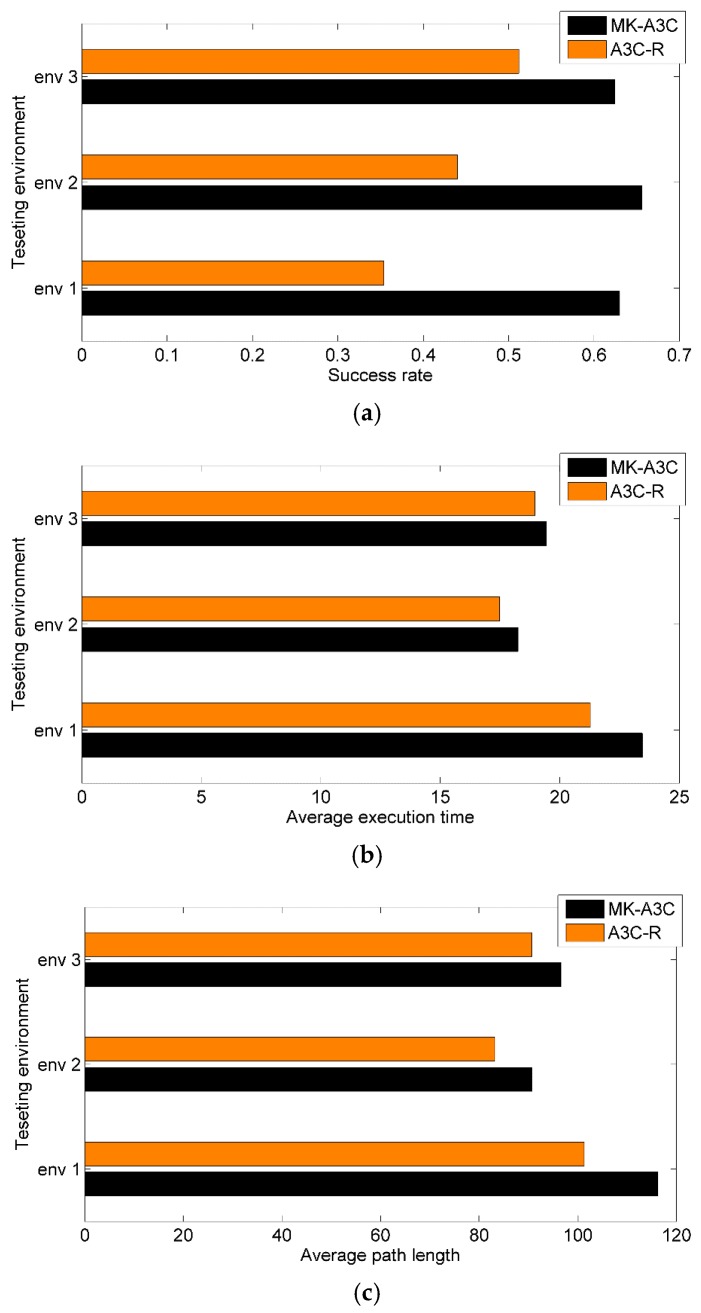
1000 navigation tasks are generated in each testing environments. (**a**) Success rate denotes the probability of successfully arriving at the destination; (**b**) Average path length denotes the mean path lengths for all successful navigation; (**c**) Average execution time denotes the average value of execution time for all successful navigation.

As shown in Figure 8a, MK-A3C achieves success rates of over 62.4% in all testing environments, whose highest success rate is 65.6% in testing environment 2. However, the success rates of A3C-R are lower than MK-A3C in all environments, especially in testing environment 1 only reaching 35.4%. Given that A3C-R plans motion behaviors only based on the current observation and ignores hidden states of the environment, it is difficult for A3C-R to deal with complex terrains and moving obstacles. Comparatively, MK-A3C integrates historical trajectories within a certain length with the current observation to estimate the complete environmental state by the GRU-based memory network, which can avoid local minima areas and handle moving obstacles. In Figure 8b,c, compared with A3C-R, navigation policies generated by MK-A3C require longer path lengths and more time to complete navigation tasks, which shows that the robot controlled by MK-A3C are more cautious about unknown environments.

## 5. Conclusions

In this paper, we propose a novel DRL algorithm called MK-A3C, which can navigate the non-holonomic robot in unknown and complex environments with moving obstacles. To solve challenges of partially observable states and sparse reward, MK-A3C makes improvements to the original A3C with memory mechanism, domain knowledge and transfer learning. Firstly, MK-A3C takes the time dimension into consideration, and constructs a GRU-based memory network architecture to strength the temporal reasoning ability of the robot. Additionally, robots with certain memory ability endowed by MK-A3C can avoid local minima traps by estimating the environmental model. Secondly, MK-A3C combines the domain knowledge-based reward function and the transfer learning-based training task architecture, which can solve non-convergence problem for policies caused by sparse reward. These improvements of MK-A3C enable controlled robots to efficiently navigate in unknown complex environments, meanwhile satisfying kinetic constraints and dealing with moving obstacles.

In the simulation experiment, first, the effectiveness of improved components of MK-A3C is verified by ablation study. These experimental results show that the GRU-based memory network can improve the performance in complex navigation tasks. The domain knowledge-based reward function and the transfer learning-based learning task architecture are essential to train useful policies. Second, the performance of MK-A3C is evaluated in unknown challenging environments with moving obstacles. Compared with A3C-R which has no memory mechanism with the same other components as MK-A3C, our method can navigate the robot in new unknown environments with high success rates.

In future work, we will consider more complex environments. Specifically, delayed actions, irregular moving obstacles and the like can be added into existing environments, which can realistically simulate a real world. In addition, this paper uses the domain knowledge-based reward function to overcome negative effects of sparse rewards, but it is time-consuming to adjust parameters of the reward function and the design of this kind reward function depends on subjective experience. It is promising to use hierarchical reinforcement learning [38], auxiliary tasks [14] and intrinsic rewards [26] to replace domain knowledge-based reward functions, which can avoid heavy work of designing reward function and further improve the training efficiency.

## Figures and Tables

**Table 1:** The first part of learning task based on transfer learning.

Training Episodes	Distance	Number of Obstacles
3000	3	1
4000	5	2
5000	9	2
3000	13	2

## References

[1] Robertson G., Watson I. (2014). A Review of Real-Time Strategy Game AI. AI Mag..

[2] Hoy M., Matveev A.S., Savkin A.V. (2015). Algorithms for collision-free navigation of mobile robots in complex cluttered environments: A survey. Robotica.

[3] Savkin A.V., Wang C. (2014). Seeking a path through the crowd: Robot navigation in unknown dynamic environments with moving obstacles based on an integrated environment representation. Robot. Auton. Syst..

[4] Uras T., Koenig S., Hernandez C. Subgoal graphs in for optimal pathfinding in eight-neighbour grids. Proceedings of the 23rd International Conference on Automated Planning and Scheduling (ICAPS ’13).

[5] Uras T., Koenig S. Identifying hierarchies for fast optimal search. Proceedings of the Twenty-Eighth AAAI Conference on Artificial Intelligence.

[6] Kavraki L.E., Švestka P., Latombe J.C., Overmars M.H. (1994). Probabilistic roadmaps for path planning in high-dimensional configuration spaces. IEEE Trans. Robot. Autom..

[7] Lavalle S.M. Rapidly-exploring random trees: Progress and prospects. Proceedings of the 4th International Workshop on Algorithmic Foundations of Robotics.

[8] Bulitko V., Lee G. (2006). Learning in real-time search: A unifying framework. J. Artif. Intell. Res..

[9] Lam C.P., Chou C.T., Chiang K.H., Fu L.C. (2011). Human-Centered Robot Navigation—Towards a Harmoniously Human–Robot Coexisting Environment. IEEE Trans. Robot..

[10] Van Den Berg J., Guy S.J., Lin M., Manocha D. (2011). Reciprocal nbody collision avoidance. Robotics Research.

[11] Tai L., Liu M. (2016). Deep-learning in Mobile Robotics—From Perception to Control Systems: A Survey on Why and Why not. arXiv.

[12] Li Y. (2017). Deep Reinforcement Learning: An Overview. arXiv.

[13] Mnih V., Badia A.P., Mirza M., Graves A., Lillicrap T., Harley T., Silver D., Kavukcuoglu K. Asynchronous methods for deep reinforcement learning. Proceedings of the International Conference on Machine Learning.

[14] Jaderberg M., Mnih V., Czarnecki W.M., Schaul T., Leibo J.Z., Silver D., Kavukcuoglu K. Reinforcement Learning with Unsupervised Auxiliary Tasks. Proceedings of the 5th International Conference on Learning Representations.

[15] Mirowski P., Pascanu R., Viola F., Soyer H., Ballard A.J., Banino A., Denil M., Goroshin R., Sifre L., Kavukcuoglu K. (2016). Learning to Navigate in Complex Environments. arXiv.

[16] Gu S., Holly E., Lillicrap T.P., Levine S. Deep reinforcement learning for robotic manipulation with asynchronous off-policy updates. Proceedings of the 2017 International Conference on Robotics and Automation.

[17] Mirowski P., Grimes M., Malinowski M., Hermann K.M., Anderson K., Teplyashin D., Simonyan K., Zisserman A., Hadsell R. (2018). Learning to Navigate in Cities Without a Map. arXiv.

[18] Cho K., Van Merrienboer B., Gulcehre C., Bahdanau D., Bougares F., Schwenk H., Bengio Y. (2014). Learning Phrase Representations using RNN Encoder-Decoder for Statistical Machine Translation. arXiv.

[19] Ng A.Y., Harada D., Russell S.J. Policy Invariance under Reward Transformations: Theory and Application to Reward Shaping. Proceedings of the International Conference on Machine Learning.

[20] Pan S.J., Yang Q. (2010). A Survey on Transfer Learning. IEEE Trans. Knowl. Data Eng..

[21] Xu H., Yu F., Darrell T., Gao Y. End-to-End Learning of Driving Models from Large-Scale Video Datasets. Proceedings of the Conference on Computer Vision and Pattern Recognition.

[22] Tai L., Li S., Liu M. A deep-network solution towards model-less obstacle avoidance. Proceedings of the 2016 IEEE/RSJ International Conference on Intelligent Robots and Systems (IROS).

[23] Agrawal P., Nair A., Abbeel P., Malik J., Levine S. Learning to Poke by Poking: Experiential Learning of Intuitive Physics. Proceedings of the 30th International Conference on Neural Information Processing Systems.

[24] Tai L., Paolo G., Liu M. Virtual-to-real deep reinforcement learning: Continuous control of mobile robots for mapless navigation. Proceedings of the 2017 IEEE/RSJ International Conference on Intelligent Robots and Systems (IROS).

[25] Wang C., Wang J., Shen Y., Zhang X. (2019). Autonomous Navigation of UAVs in Large-Scale Complex Environments: A Deep Reinforcement Learning Approach. IEEE Trans. Veh. Technol..

[26] Zhelo O., Zhang J., Tai L., Liu M., Burgard W. (2018). Curiosity-driven Exploration for Mapless Navigation with Deep Reinforcement Learning. arXiv.

[27] Canny J. (1988). The Complexity of Robot Motion Planning.

[28] Lillicrap T.P., Hunt J.J., Pritzel A., Heess N., Erez T., Tassa Y., Silver D., Wierstra D. (2015). Continuous control with deep reinforcement learning. Comput. Sci..

[29] Schulman J., Wolski F., Dhariwal P., Radford A., Klimov O. (2017). Proximal Policy Optimization Algorithms. arXiv.

[30] Heess N., Tb D., Sriram S., Lemmon J., Merel J., Wayne G., Tassa Y., Erez T., Wang Z., Eslami S.M.A. (2017). Emergence of Locomotion Behaviours in Rich Environments. arXiv.

[31] Sutton R.S., Barto A.G. (2018). Reinforcement Learning: An Introduction.

[32] Mnih V., Kavukcuoglu K., Silver D., Rusu A., Veness J., Bellemare M.G., Graves A., Riedmiller M., Fidjeland A.K., Ostrovski G. (2015). Human-level control through deep reinforcement learning. Nature.

[33] Van Hasselt H., Guez A., Silver D. (2015). Deep Reinforcement Learning with Double Q-learning. arXiv.

[34] Hessel M., Modayil J., Van Hasselt H., Schaul T., Ostrovski G., Dabney W., Horgan D., Piot B., Azar M., Silver D. Rainbow: Combining Improvements in Deep Reinforcement Learning. Proceedings of the National Conference on Artificial Intelligence.

[35] Schulman J., Levine S., Moritz P., Jordan M.I., Abbeel P. Trust region policy optimization. Proceedings of the International Conference on Machine Learning.

[36] Bengio Y., Louradour J., Collobert R., Weston J. Curriculum learning. Proceedings of the 26th Annual International Conference on Machine Learning, (ICML 2009).

[37] Zaremba W., Sutskever I. (2014). Learning to Execute. arXiv.

[38] Vezhnevets A., Osindero S., Schaul T., Heess N., Jaderberg M., Silver D., Kavukcuoglu K. FeUdal networks for hierarchical reinforcement learning. Proceedings of the 2017 International Conference on Machine Learning.

